# Evidenced-Based Approaches to Support the Development of Endocrine-Mediated Adverse Outcome Pathways: Challenges and Opportunities

**DOI:** 10.3389/ftox.2021.787017

**Published:** 2021-12-21

**Authors:** Karine Audouze, Elias Zgheib, Khaled Abass, Asma H. Baig, Isabel Forner-Piquer, Henrik Holbech, Dries Knapen, Pim E. G. Leonards, Diana I. Lupu, Saranya Palaniswamy, Arja Rautio, Maria Sapounidou, Olwenn V. Martin

**Affiliations:** ^1^ Université de Paris, T3S, Inserm U1124, Paris, France; ^2^ Thule Institute, University of Arctic, University of Oulu, Oulu, Finland; ^3^ Department of Pesticides, Menoufia University, Menoufia, Egypt; ^4^ Centre for Pollution Research and Policy, Brunel University London, Uxbridge, United Kingdom; ^5^ Department of Biology, University of Southern Denmark, Odense, Denmark; ^6^ Zebrafishlab, Department of Veterinary Sciences, University of Antwerp, Wilrijk, Belgium; ^7^ Department of Environment and Health, Vrije Universiteit Amsterdam, Amsterdam, Netherlands; ^8^ Evolutionary Biology Centre, Uppsala University, Uppsala, Sweden; ^9^ Center for Life Course Health Research, Faculty of Medicine, University of Oulu, Oulu, Finland; ^10^ Department of Chemistry, Faculty of Science and Technology, Umeå University, Umeå, Sweden

**Keywords:** adverse outcome pathways, endocrine disruption, systematic (literature) review, machine learning, evidence-based methods

## Introduction

A transformation of regulatory toxicology is underway to meet the demands of testing increasing numbers of chemicals whilst reducing reliance on *in vivo* models. This transformation requires a shift from chemical safety assessment largely based on direct empirical observation of apical toxicity outcomes in whole organisms to predictive approaches in which outcomes and risks are inferred from accumulated mechanistic understanding. In the last decade, Adverse Outcome Pathways (AOPs) (Ankley et al., 2010; Ankley and Edwards, 2018) have captured the attention of regulators and researchers alike as a systematic approach for organizing knowledge that may support such inferences (Wittwehr et al., 2017).

An AOP is a conceptual structured representation of existing toxicological knowledge describing the causally connected sequence of events, across different levels of biological organization, required to produce an adverse effect when an organism is exposed to a stressor. Specifically, AOPs depict a series of key events (KEs) linking a molecular initiating event (MIE, an interaction between a stressor (*e.g.*, endogenous ligand, xenobiotic) and a biomolecule) to an adverse outcome (AO, at organism or population levels). The causal links between 2 KEs are referred to as key event relationships (KERs). AOPs provide a useful framework to connect mechanistic data to adverse effects on human health or wildlife populations as a basis for the identification of cell- or biochemical-based tests that could fit in Integrated Approaches to Testing and Assessment (IATAs), identifying KEs that could be targeted for the development of New Approach Methods (NAMs), as well as investigating similarities in mechanistic pathways between species.

AOPs are also particularly salient for identifying potential Endocrine Disruptors (EDs). Indeed, both the World Health Organization (WHO) and the International Programme on Chemical Safety (IPCS) definition and the scientific criteria adopted by the European Union in 2017 are articulated around three key requirements, namely; evidence of an adverse effect, evidence of an endocrine-mediated (EM) mode-of-action, and the plausibility of the causal relationship between the mode-of-action and adverse effect. Both the European Chemical Agency (ECHA) and European Food Safety Authority (EFSA) recommend that more EM AOPs be developed for substantiation of chemicals’ ED properties [European Chemical Agency (ECHA) et al., 2018]. Of the 370 AOPs currently included in the AOP-wiki (aopwiki.org), there are 80 EM AOPs (Table 1).

**TABLE 1:** EM AOPs in AOP-wiki.

AOP title	Id	Author status			Saaop status	
		Under development	Open for citation and comment	Open for adoption	Under development (outside of OECD work Plan)	Included in OECD work plan
		52	18	8	24	35 (Dev: 14, Rev: 13, Apr: 1, End: 7)
Related to androgen axis (15 AOPs)						
PPAR*α* activation *in utero* leading to impaired fertility in males	18		x			EAGMST Under review (Rev.)
Androgen receptor antagonism leading to adverse effects in the male foetus (mammals)	19			x	x	
Androgen receptor agonism leading to reproductive dysfunction (in repeat-spawning fish)	23		x			WPHA/WNT Endorsed (End.)
Decrease in androgen receptor activity leading to Leydig cell tumors (in rat)	111	x			x	
Androgen receptor activation leading to hepatocellular adenomas and carcinomas (in mouse and rat)	117			x		Under development (Dev.)
HMG-CoA reductase inhibition leading to decreased fertility	124	x			x	
Inhibition of 17*α*-hydrolase/C 10,20-lyase (Cyp17A1) activity leads to birth reproductive defects (cryptorchidism) in male (mammals)	288		x			
5*α*-reductase inhibition leading to short anogenital distance (AGD) in male (mammalian) offspring	305	x				Dev
Androgen receptor (AR) antagonism leading to short anogenital distance (AGD) in male (mammalian) offspring	306					Dev
Decreased testosterone synthesis leading to short anogenital distance (AGD) in male (mammalian) offspring	307	x				Dev
Androgen receptor (AR) antagonism leading to nipple retention (NR) in male (mammalian) offspring	344	x				
Androgen receptor (AR) antagonism leading to decreased fertility in females	345	x				
Inhibition of 11*β*-Hydroxysteroid Dehydrogenase leading to decreased population trajectory	348	x				
Androgen receptor antagonism leading to testicular cancer	372	x				
Androgen receptor agonism leading to male-biased sex ratio	376	x				
Related to estrogen axis (24 AOPs)						
Aromatase inhibition leading to reproductive dysfunction	25		x			End
Estrogen receptor agonism leading to reproductive dysfunction	29	x			x	
Estrogen receptor antagonism leading to reproductive dysfunction	30		x			Rev
Sustained AhR Activation leading to Rodent Liver Tumours	41		x			Rev
Constitutive androstane receptor activation leading to hepatocellular adenomas and carcinomas in the mouse and the rat	107		x			Rev
Increased dopaminergic activity leading to endometrial adenocarcinomas (in Wistar rat)	112	x			x	
Aryl hydrocarbon receptor activation leading to uroporphyria	131		x			End
Endocytic lysosomal uptake leading to liver fibrosis	144	x				Rev
Estrogen Receptor Activation and Female Precocious Puberty	146	x			x	
Aryl hydrocarbon receptor activation leading to early life stage mortality, *via* reduced VEGF	150		x			End
Antiestrogen activity leading to ovarian adenomas and granular cell tumors in the mouse	165	x			x	
Early-life estrogen receptor activity leading to endometrial carcinoma in the mouse.	167	x			x	
Estrogen receptor activation leading to breast cancer	200			x	x	
Cyp2E1 Activation Leading to Liver Cancer	220		x			End
Deposition of energy leading to lung cancer	272	x				Rev
Inhibition of 5*α*-reductase leading to impaired fecundity in female fish	289		x			Dev
Increased DNA damage leading to increased risk of breast cancer	293	x				Dev
Increased reactive oxygen and nitrogen species (RONS) leading to increased risk of breast cancer	294	x				Dev
Early-life stromal estrogen receptor activation by endocrine disrupting chemicals in the mammary gland leading to enhanced cancer risk	295	x				Dev
Binding to estrogen receptor (ER)-*α* in immune cells leading to exacerbation of systemic lupus erythematosus (SLE)	314	x				Dev
Glucocorticoid Receptor Agonism Leading to Impaired Fin Regeneration	334	x				
Aromatase inhibition leads to male-biased sex ratio *via* impacts on gonad differentiation	346	x				
Decreased fibrinolysis and activated bradykinin system leading to hyperinflammation	392	x				
G protein-coupled estrogen receptor 1 (GPER) signal pathway in the endocrine disrupting effect	401	x				
Related to thyroid axis (31 AOPs)						
Aromatase (Cyp19a1) reduction leading to impaired fertility in adult female	7		x			Rev
Upregulation of Thyroid Hormone catabolism *via* Activation of Hepatic Nuclear Receptors, and Subsequent Adverse Neurodevelopmental Outcomes in Mammals	8	Not under active development		Dev		
Protein Alkylation leading to Liver Fibrosis	38		x		x	
Inhibition of Thyroperoxidase and Subsequent Adverse Neurodevelopmental Outcomes in Mammals	42		x			End
Inhibition of Na+/I- symporter (NIS) leads to learning and memory impairment	54		x			End
Inhibition of iodide pump activity leading to follicular cell adenomas and carcinomas (in rat and mouse)	110	x			x	
Inhibition of thyroid peroxidase leading to follicular cell adenomas and carcinomas (in rat and mouse)	119	x			x	
Kidney dysfunction by decreased thyroid hormone	128	x				Dev
Sodium Iodide Symporter (NIS) Inhibition and Subsequent Adverse Neurodevelopmental Outcomes in Mammals	134	x			x	
Interference with thyroid serum binding protein transthyretin and subsequent adverse human neurodevelopmental toxicity	152	x				Dev
Deiodinase 2 inhibition leading to increased mortality *via* reduced posterior swim bladder inflation	155			x		Rev
Deiodinase 2 inhibition leading to increased mortality *via* reduced anterior swim bladder inflation	156			x		Rev
Deiodinase 1 inhibition leading to increased mortality *via* reduced posterior swim bladder inflation	157			x		Rev
Deiodinase 1 inhibition leading to increased mortality *via* reduced anterior swim bladder inflation	158			x		Rev
Thyroperoxidase inhibition leading to increased mortality *via* reduced anterior swim bladder inflation	159			x		Rev
Enhanced hepatic clearance of thyroid hormones leading to thyroid follicular cell adenomas and carcinomas in the rat and mouse	162	x			x	
Thyroperoxidase inhibition leading to altered amphibian metamorphosis	175	x			x	
Sodium Iodide Symporter (NIS) Inhibition leading to altered amphibian metamorphosis	176	x			x	
Iodotyrosine deiodinase (IYD) inhibition leading to altered amphibian metamorphosis	188	x			x	
Type I iodothyronine deiodinase (DIO1) inhibition leading to altered amphibian metamorphosis	189	x			x	
Type II iodothyronine deiodinase (DIO2) inhibition leading to altered amphibian metamorphosis	190	x			x	
Type III iodotyrosine deiodinase (DIO3) inhibition leading to altered amphibian metamorphosis	191	x			x	
Inhibition of thyroid peroxidase leading to impaired fertility in fish	271	x				Dev
Oxidative DNA damage leading to chromosomal aberrations and mutations	296		Open for comment. Do not cite			Rev
Thyroid Receptor Antagonism and Subsequent Adverse Neurodevelopmental Outcomes in Mammals	300	x				Dev
Thyroperoxidase inhibition leading to increased mortality *via* altered retinal layer structure	363	x				
Competitive binding to thyroid hormone carrier protein transthyretin (TTR) leading to altered amphibian metamorphosis	366	x				
Competitive binding to thyroid hormone carrier protein thyroid binding globulin (TBG) leading to altered amphibian metamorphosis	367	x				
AOP for thyroid disorder caused by triphenyl phosphate	393	x				
Inhibition of Fyna leading to increased mortality *via* decreased eye size (Microphthalmos)	399	x				
Thyroid peroxidase (TPO) inhibition leads to periventricular heterotopia formation in the developing rat brain	402	x				
Other (10 AOPs)						
Ecdysone receptor agonism leading to incomplete ecdysis associated mortality	4		x		x	
GnRH pulse disruption leading to pituitary adenomas and carcinomas in the SD rat	169	x			x	
Anti-dopaminergic activity leading to mammary adenomas and carcinomas in the SD rat	170	x			x	
Type I iodothyronine deiodinase (DIO1) inhibition leading to altered amphibian metamorphosis	189	x			x	
Juvenile hormone receptor agonism leading to male offspring induction associated population decline	201	x			x	
Luteinizing hormone receptor antagonism leading to reproductive dysfunction	309	x				
PPARalpha Agonism Impairs Fish Reproduction	323	x				
Inhibition of 11*β*-hydroxylase leading to decreased population trajectory	349	x				
Chitin synthase 1 inhibition leading to mortality	360		x			EAGMST approved (Apr.)
Photosystem II antagonism leading to growth inhibition *via* dysregulation of growth hormone	371	x				

Evidence-Based Methodologies (EBMs), including systematic maps and systematic reviews (SRs), aim to help assemble and assess the current knowledge on a topic in an objective, comprehensive and transparent manner. Although the AOP development process does not currently explicitly require use of EBMs (De Vries et al., 2021), efforts are ongoing to recommend AOP developers to document the most important aspects of their work, including the overall data identification, screening and evaluation strategy. EBMs adapted to AOP development, are desirable to increase transparency and promote the re-use of AOP components and their underlying data, and to inform the use for specific applications by the regulators. Nevertheless, these advantages need to be balanced with time and effort involved in their implementation. There is therefore an urgent need to further develop automated or semi-automated Machine Learning Tools (MLT) that can assist with specific steps of systematic evidence retrieval and assessment.

This perspective article draws on lessons learnt from the EURION cluster activities to review the circumstances in which EBMs approaches may be most usefully applied to EM AOP development and opportunities for further research and development of tools tailored to mechanistic evidence gathering and evaluation.

## EURION Activities Relevant to AOP Development

EURION is a cluster of eight research projects, funded under the European Commission’s Horizon 2020 Research and Innovation Programme, focusing on developing new testing and screening methods identifying EDs and studying their effects on metabolism (Audouze et al., 2020; Küblbeck et al., 2020; Legler et al., 2020), female reproductive toxicity (van Duursen et al., 2020), developmental neurotoxicity (Lupu et al., 2020) and thyroid-mediated toxicity (Holbech et al., 2020; Kortenkamp et al., 2020; Moroni et al., 2020). AOP development is an integral part of each of these projects (Street et al., 2021).

However, limited availability of test methods assessing EDs leave significant knowledge gaps in identification of EDs properties (European Commission. Joint Research Centre, 2017), that can be highlighted through modelling of underlying mechanisms of action and delineation of AOPs. In some cases, the MIE is known and endpoints can be experimentally observed. For example, in the thyroid hormone system, inhibition of thyroid peroxidase (involved in thyroid hormones (TH) production) has been associated with the formation of periventricular heterotopia, symptomatic disruption of thyroid-dependent neuronal migration (Ramhøj et al., 2021), yet not currently adequately investigated by available test guidelines (Kortenkamp et al., 2020).

AOP development within the EURION cluster is ongoing for different modalities, particularly, those related to androgen, thyroid hormone, and retinoid signalling. An AOP working group (WG) has been set up to support and facilitate AOP-related activities within the EURION projects and to bring together AOP-structured information and data across projects. This WG has identified potential synergies and areas for collaboration among projects for which EBMs may be relevant.

Collaborative AOP development could be supported by mapping the underlying canonical knowledge on the general biology of the endocrine system as well as relevant well-established pathologies. In particular, it may be helpful to uncouple the development of downstream KER descriptions, which are related to general/apical organismal effects or processes that can be described based on canonical knowledge and are relevant to many different AOPs, from the description of upstream mechanistic KERs that are often highly specific to a particular toxicological context. Further, KEs and KERs may be shared by several AOPs, forming AOP networks. Linear pathways can converge and diverge several times (Knapen et al., 2018) producing crossover nodal points in the network. An example is decreased TH, which is involved in AOs in foetal neurodevelopment irrespective of the MIE. KE or KER descriptions therefore emerge as promising units of collaborative AOP development uncoupled from the MIE (Svingen et al., 2021). Since KERs are putatively causally-related pairs of events, they are amenable to evaluation in a similar way to an exposure-outcome pair conventionally investigated in an EBM approach (*i.e.*, SRs). A potential application of EBM in AOP development may therefore be as a series of KERs’ SRs (De Vries et al., 2021).

## Evidence-Based Protocol

A central premise of EBMs is that protocols be developed and published or registered *a priori* to avoid introducing bias. Applying a similar requirement to the development of AOPs ought to consider the iterative nature of AOP development and focus on identifying KERs most amenable to systematically gathering and assessing existing literature.

### Establishing *a Priori* Biological Plausibility

Whilst the nature of KERs allows them to be “good” EBMs products, some good practices in their conception would make them even more SR-compatible. Whether for the construction of an AOP *de novo*, or for the addition of KEs to an existing AOP, KERs should be defined with enough specificity to give AOP users a clear idea of measurable objects at each step of the AOP state. Ideally, a clear and transparent rationale for the consideration of a specific KER ought to be established *a priori*. This requires integrating knowledge beyond the realm of regulatory toxicology, and this point is particularly relevant for EM pathways. Not only is a grasp of canonical knowledge a prerequisite but it should also be completed by evidence of medical conditions such as known hormonal imbalances or genetic defects in proteins (*e.g.*, receptors, enzymes), as well as the physiology and signalling pathways of endogenous ligands or well-characterised (pharmacological) agonists or antagonists especially relevant to the KE of interest. For instance, the Allan-Herndon-Dudley syndrome is caused by a rare defect in the gene encoding for monocarboxylate transporter 8 (MCT8) that leads to severe brain development deficits (impaired TH transport across brain cell membranes). Literature related to clinical presentation and putative pharmacological interventions (Grijota-Martínez et al., 2020), as well as evidence of the existence of xenobiotics capable of inhibiting MCT8 (Johannes et al., 2016), represent important contextual information when considering the biological plausibility of inhibition of triiodothyronine (T3) uptake by MCT8 as a potential MIE. It is therefore clear that a substantial review effort has to take place during problem formulation (PF) prior to the formulation of objectives. This may take the form of a systematic evidence map, wider in scope than a SR, the results of which could then form the basis of more targeted SRs of the evidence in relation to knowledge gaps identified.

### Problem Formulation and Applicability Domain

PF is a crucial first step for the development of both a proposed AOP and protocol for EBMs. The issue presented here concerns the structured terms used to define a specific context that narrows the applicability of the KER, and the universality of the AOP. This process mirrors that of developing PECO (Population, Exposure, Comparator, Outcome) or similar statements used in EBMs. One single KER should avoid compounding events together and rather refer to one single measurable event (OECD, 2018). For instance, using the expression “fatty acid beta oxidation” is more precise and useful for EBMs, especially SRs, than the broad statement “lipid metabolism”. It is easier to retrieve evidence concerning specific KEs measured with reliable and relevant methods, than to investigate broad and general phenomena. Specificity and precision have the advantage of making a KER more searchable. Notably, an artificial intelligence based approach using text mining and graph theory, named AOP-helpFinder, allows to automatically identify and extract specific AOP-related terms and evidence (aop-helpfinder.u-paris-sciences.fr) (Jornod et al., 2020; Jornod et al., 2021; Zgheib et al., 2021). In “Rugard et al., 2020”, AOP-helpFinder results pointed out linkages between Bisphenol-F and relevant MIEs [*i.e.*, “PPAR*γ* inhibition” (Peroxisome proliferator-activated receptor γ)] often associated with liver steatosis and lipid accumulation (Yang et al., 2010).

Another key parameter is the biological domain of applicability (e*.g.*, taxonomy, sex, species, life stage). In theory, an AOP’s relevant biological domain of applicability is restricted to the narrowest domain of its components. For a specific KER, query terms should remain inclusive as long as the observed biological object (*e.g.*, protein, organ) and its function are conserved across the domain category. However, restriction of the biological context becomes a must when equivalency of the structure or the function are not preserved. Typically, translational considerations should take into account the relevance of predicting AOs in humans from experimental animal models. For instance, certain female reproductive endpoints (*e.g.*, menstruation, endometriosis, placentation) in humans are absent or different in rodents, and vice versa (*e.g.*, aromatase inhibition caused dystocia) (Bruner-Tran et al., 2018). This emphasises the importance of evolutionary biology and comparative endocrinology. Concretely, if a KER involves binding to a specific nuclear receptor, but a certain species lacks a functional homolog of that receptor, the AOP would not be relevant to that species on the basis of a lack of conserved structure. Likewise, KERs involving measurements in ovary tissue would be restricted to females, and those concerning hepatic enzymes would be reserved to liver-inclusive AOPs (OECD, 2018). For example, in their study of 2016, “Schulz and Sisk, 2016” displayed an interesting example of sex- and life-specific organizational effects of sex steroids on reproductive behaviour during brain development. Bioinformatics and sequence similarity approaches have been adapted and employed for AOPs of human health and environment (LaLone et al., 2016, 2013). LaLone et al. (2018) showcased the use of sequence alignment to predict across species susceptibility (SeqAPASS) for species extrapolation from high-throughput screening results including case studies on evidence for conservation of certain endocrine targets across vertebrate species.

### Search Strategies

The design of adequately sensitive and sufficiently specific systematic literature search strategies for AOP development, particularly when a KE is used as a starting point, is challenging. AOPs are assumed to be chemical agnostic, *i.e.*, their applicability should not be limited to a specific substance. However, search strategies employed in EBMs are typically articulated around search terms related to both the stressor (exposure) and the outcome (endpoint). The chemical agnosticism of AOPs raises questions over the validity of literature search strategies referring to specific substances. Nevertheless, the evidence used to support each KER is based on chemical-specific exposure data (Leist et al., 2017). However, since in reality we are usually simultaneously exposed to mixtures of exposures and rarely to single chemicals, carrying on multiple chemical-specific search analyses, concerning different stressors, and combining their results, might pull out, at least partially, the approach from chemical-specificity towards chemical-agnosticism again. Conversely, many chemical stressors can putatively interfere with one or several AOPs and this is another case where combination of search strategies would be relevant for regulatory decision-making (Aguayo-Orozco et al., 2019).

These facts unveil the complexity of interactions in biology and allow to detect a balance point for the adequate approach between chemical-independency and chemical-specificity. Therefore, search strategies using generic terms instead of names of specific substances may be too unspecific whilst not guaranteeing sensitivity. However, although the generic terms produce a large number of hits, when combined with carefully thought out terms for other parts of the question (*e.g.*, biological targets), they help search tools cover a larger spectrum. To our knowledge, this aspect has not yet been systematically investigated. One approach may be to develop search strategies for prototypical stressors or model compounds chosen from reliable resources like EURL ECVAM (European Union Reference Laboratory, European Centre for the Validation of Alternative Methods), for the methods/models validation (Sund and Deceuninck, 2021).

Another approach to support the development of adequate search strategies would be to evaluate *a priori* the chemical applicability domain of specific KEs. A proof of concept used the case of ‘PPARγ dysregulation’ to characterize the chemical domain of PPARγ full agonists (Al Sharif et al., 2017). Approaches for chemical screening to capture MIEs relevant to human health have been proposed for a multitude of human relevant targets by the Danish database (qsar.food.dtu.dk/), for hepatic steatosis (Gadaleta et al., 2018) and for targets of pharmaceutical relevance (Allen et al., 2016). Similarly, attempts for chemical screening across environmental species and targets relevant to aquatic toxicology have been also proposed (Bauer et al., 2018; Sapounidou et al., 2021). A notable artificial intelligence based *in silico* approach called AOP4EUpest (biomedicale.parisdescartes.fr/aop4EUpest/home.php), is a web server that has been developed to employ text-mining and graph theory approaches for identification of literature that links specific pesticides with AOP events.

Further research is necessary to establish to what extent devising search strategies including terms related to specific chemical stressors would be suitable to retrieve negative evidence and whether they may compound publication bias or limit the applicability domain of an AOP. Such research should serve to develop a set of recommendations for literature search algorithms adapted to the retrieval of EM mechanistic data for AOP development.

In addition, development of AOPs should be seen as an interdisciplinary framework, as it involves different and complementary expertise (toxicologists, biochemists, bioinformatics scientists, etc.), in order to decipher the most complete picture of the biological complexity of systems.

### Critical Appraisal

**FIGURE 1:**
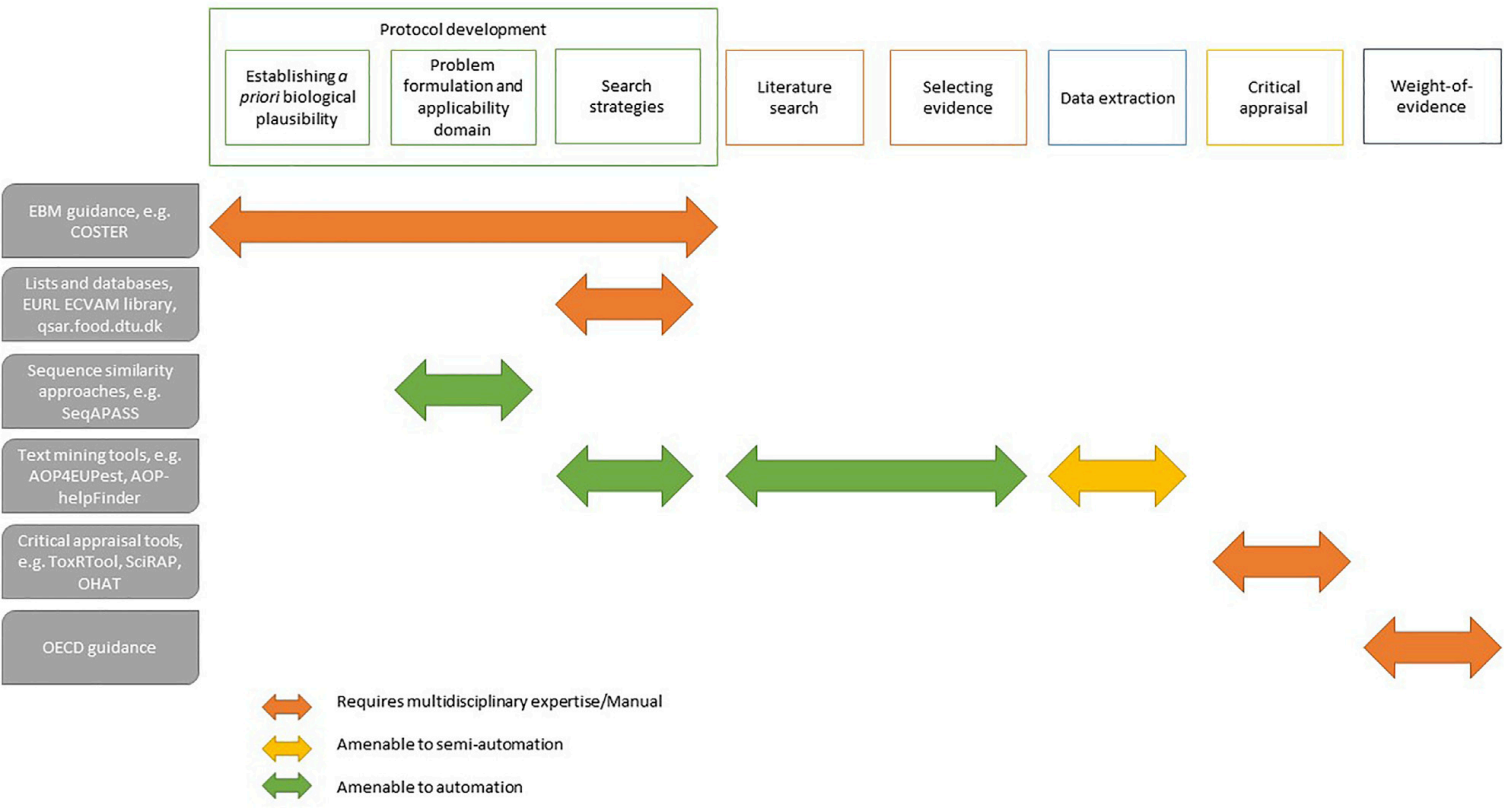
Overview of EBM processes for KER development amenable to automation with illustrative supporting tools and resources.

Some critical appraisal tools of the risk-of-bias of individual studies are commonly used for risk assessment (*e.g.*, ToxRTool. ec.europa.eu/jrc/en/scientific-tool/toxrtool-toxicological-data-reliability-assessment-tool), and some have been adapted to cater for the appraisal like the SciRAP protocol (Roth et al., 2021). ToxRTool provides comprehensive criteria and guidance for evaluations of the inherent quality of data, making the reliability evaluation process of assigning reliability categories more transparent and harmonised. Automation doesn’t apply to these tools yet nor would in the foreseeable future (Figure 1). Therefore, the focus at this stage should fall on the expertise of the evaluator and the inter-rater protocol that is followed. Simple approaches based on formulating clearly framed questions (*i.e.*, decision tree) may be of help not only for critical appraisal but also for other aspects of the process, like PF (Morgan et al., 2018). This conceptual modelling is a transparent and coherent approach that facilitates the integration and interpretation of knowledge across the whole SR process (Roth et al., 2020).

### Weight-Of-Evidence

Similarly when it comes to integrating and synthesizing the overall body of evidence, WoE approaches developed specifically to assess whether specific substances are EDs and are not adapted to establish confidence in individual KERs or the biological plausibility of AOPs (Vandenberg et al., 2016). The OECD guidance document describes relevant criteria and methods for both the WoE for KERs and overall assessment of the AOPs that can be equally applied to systematically retrieved evidence (Becker et al., 2015). This existing guidance is aligned with the work of Meek et al. (2014) and adapts Bradford-Hill considerations to the specific context of assessing and integrating mechanistic evidence. Again, applications of this WoE method invariably rely on expert judgment and emphasis should be here on documenting the process and rationale transparently, and it is questionable whether automation of the process is even desirable.

## Closing Remarks

It is recognized that many of the EM AOPs that are currently available in the AOP-wiki are incomplete, and often remain in that state for a relatively long period of time. Indeed, only 26 of the 80 AOPs listed in Table 1, or about 33%, are considered either “Open for adoption” or “Open for citation or comment” by their authors, and only seven AOPs (9%) are OECD endorsed. Overall, it is clear that there is a need to incentivize and encourage completion of initiated AOPs. First, development of an AOP can be perceived as a daunting task, requiring the knowledge and evidence to describe the different KEs and KERs, including establishing causality and essentiality, documenting dose and time concordance, etc. Second, many AOP authors, in particular those from academia, feel that there is insufficient professional recognition for the scholarly investment that is required for formal AOP development, review and endorsement (Knapen, 2021; Svingen et al., 2021). A number of initiatives are being taken to address these challenges. For example, AOP authors who add their AOP under development to the OECD work plan are entitled to free guidance and coaching by an AOP expert, ensuring completion of the AOP following the official OECD’s AOP development principles. Also, several journals, including Environmental Toxicology and Chemistry and Environmental and Molecular Mutagenesis, have launched a new “AOP Report” article type, allowing the publication of AOPs, individual KERs, or small AOP networks. This provides authors with a peer-reviewed publication, one of the main relevant academic output parameters worldwide. These journals have entered into an agreement with OECD allowing the accepted journal article, including the reviewed AOP, to directly enter the OECD’s endorsement process without going through the OECD’s scientific review process.

In this context, resources such as time and access to literature remain considerable barriers to the implementation of EBM to AOP development. Echoing the views expressed in Svingen et al. (2021), systematic evidence mapping may support PF in complementing canonical knowledge and identifying KERs for which SRs is appropriate. Indeed, given that EBM are often energy- and time-consuming, scientists should be selective about when a SR is needed, in order to keep the barrier to AOP development, and completion of initiated AOPs, sufficiently low.

A number of MLT that have been or are being developed can support the earlier stages of SR adapted to AOP development (*i.e.*, PF, search strategies design). Their implementation for information retrieval ought to be validated and compared with manual methods for completeness to avoid introducing potential biases. It also is unclear whether automated methods would be compatible with the demands for transparently documented appraisal of the quality of individual pieces of evidence or the evaluation strength of the overall body of evidence. The development of MLT to support evidence-based AOP development is a promising field, that is complementary to already existing approaches, but that will nonetheless require a considerable validation effort before full implementation.

## Data Availability

The original contributions presented in the study are included in the article/supplementary material, further inquiries can be directed to the corresponding author.

## Abbreviations

AO, Adverse Outcome; AOP, Adverse Outcome Pathway; EBM, Evidence-Based Methodologies; ECHA, European Chemical Agency; ECVAM, European Centre for the Validation of Alternative Methods; ED, Endocrine Disruptor; EFSA, European Food Safety Authority; EM, Endocrine-Mediated; EURL, European Union Reference Laboratory; IATA, Integrated Approaches to Testing and Assessment; IPCS, International Programme on Chemical Safety; KE, Key Event; KER, Key Event Relationship; MIE, Molecular Initiating Event; MCT8, MonoCarboxylate Transporter eight; MLT, Machine Learning Tools; NAM, New Approach Methods; PECO, Population, Exposure, Comparator and Outcome; PF, Problem Formulation; PPAR*γ*, Peroxisome proliferator-activated receptor *γ;* SeqAPASS, Sequence Alignment to Predict Across Species Susceptibility; SR, Systematic Reviews; T3, Triiodothyronine; TH, Thyroid Hormones; WG, Working Group; WHO; World Health Organization; WoE, Weight-of-Evidence.
